# Outside the Exam Room: Policies for Connecting Clinic to Community in Diabetes Prevention and Treatment

**DOI:** 10.5888/pcd12.140403

**Published:** 2015-05-07

**Authors:** Jason Q. Purnell, Cynthia Herrick, Sarah Moreland-Russell, Amy A. Eyler

**Affiliations:** Author Affiliations: Cynthia Herrick, Sarah Moreland-Russell, Amy A. Eyler, Brown School, Washington University in St Louis, St Louis, Missouri.

## Abstract

The public health burden and racial/ethnic, sex, and socioeconomic disparities in obesity and in diabetes require a population-level approach that goes beyond provision of high-quality clinical care. The Robert Wood Johnson Foundation’s Commission to Build a Healthier America recommended 3 strategies for improving the nation’s health: 1) invest in the foundations of lifelong physical and mental well-being in our youngest children; 2) create communities that foster health-promoting behaviors; and 3) broaden health care to promote health outside the medical system. We present an overview of evidence supporting these approaches in the context of diabetes and suggest policies to increase investments in 1) adequate nutrition through breastfeeding and other supports in early childhood, 2) community and economic development that includes health-promoting features of the physical, food, and social environments, and 3) evidence-based interventions that reach beyond the clinical setting to enlist community members in diabetes prevention and management.

## Introduction

Preventing and treating diabetes are major public health priorities in light of the increased risk for disability and premature death associated with the disease. Diabetes is the seventh leading cause of death in the United States, contributes to cardiovascular, renal, vision, and other complications, and results in $245 billion in total costs (1). In 2005 through 2010, an estimated 21 million adults aged 20 or older in the United States had diabetes, a 9.3% prevalence (vs 5.5% in 1988–1994 and 7.6% in 1999–2004) (2). Trends suggest that diabetes prevalence has been increasing over the past several decades in conjunction with a sharp increase in the prevalence of obesity. Racial/ethnic disparities have increased over the same period; in 2005 through 2010, prevalence among African Americans (15.4%) and Mexican Americans (11.6%) was significantly higher than prevalence among non-Hispanic whites (8.6%) (2). In 2011 through 2012, the prevalence of obesity was 8.1% among infants and toddlers, 16.9% among children and youths aged 2 to 19 years, and 34.9% among adults aged 20 years or older, with prevalence higher among adult women than men and higher among non-Hispanic blacks and Hispanics than non-Hispanic whites and non-Hispanic Asians (3). Both the considerable public health burden and the significant racial/ethnic and sex disparities in obesity and in diabetes prevalence, control, and mortality require a population-level approach that goes beyond reliance on what clinical interventions can address to reduce the burden of these conditions (4).

In January of 2014, the Robert Wood Johnson Foundation’s Commission to Build a Healthier America (hereafter referred to as the Commission) recommended 3 broad strategies for improving the nation’s health: 1) invest in the foundations of lifelong physical and mental well-being in our youngest children, 2) create communities that foster health-promoting behaviors, and 3) broaden health care to promote health outside the medical system (5). These recommendations, although not specific to any particular condition, frame the approach to diabetes prevention and treatment described in this article. Specifically, we argue that efforts to prevent obesity and diabetes must begin in the earliest years of life and should be integrated into high-quality early childhood programs. Such programs necessarily include evidence-based interventions to address nutrition in young children as the foundation for a health-promoting behavior they will continue into adolescence and adulthood. To the extent that early intervention increases educational attainment (6) and higher levels of education predict better health behaviors (7), supporting early childhood development should also have indirect effects on obesity and diabetes. Cardiovascular and metabolic disease risks were lower for individuals in their mid-30s who had received high-quality early intervention as children than for those who had not received this intervention (8).

Within neighborhoods and communities, features of the built environment (eg, community design conducive to walking), the food environment (eg, access to healthful foods), and other aspects of community and economic development (eg, jobs, housing, transportation) should support behaviors that promote obesity and diabetes prevention (9–11). As the Commission notes, “efforts should be made to improve the health of all communities, [but] we must prioritize communities where low-income Americans lack opportunities to make healthy choices” (p. 22) (5). Health care access within communities is also a necessary condition for obesity and diabetes prevention and treatment, and includes not only physical proximity but also affordability and culturally appropriate care. Although necessary, access alone is not sufficient for prevention and treatment.

In the clinical setting and at its interface with the community, there are multiple opportunities to address diabetes prevention and treatment. The emphasis on high-quality treatment and the coordination of patient care (eg, patient-centered medical homes [PCMHs]) within the Affordable Care Act has prompted a reassessment of health care delivery in the United States. Prevention and treatment of diabetes, particularly in socially disadvantaged and traditionally underserved populations, will require far greater coordination than exists now among providers and between health care systems and community-based partners. The Commission calls for an expansion of the concept of “vital signs” in clinical and public health settings to include nonmedical factors such employment, education, health literacy, and safe housing. The Commission also envisions “prescriptions” for behaviors such as healthful eating that can be “filled” with community-based programs (5).

We provide an overview of the evidence for each of these approaches. We also present a model for how policies can be enacted that will bridge the clinic and the community in diabetes prevention and treatment efforts, while noting examples of best practices. Finally, we will highlight future directions for research, practice, and policy in this area.

## Invest in the Foundations of Lifelong Physical and Mental Well-Being in Our Youngest Children

Poor early childhood nutrition can negatively affect children’s physical and emotional development; it can increase their risk for obesity and diabetes and limit their adult achievement and productivity (12). There are many factors that affect children’s nutrition, including those related to social, familial, cultural, and community influences. Research shows that the first 3 years of life are a period of rapid brain development and physical growth (13). Consequently, without proper nutrition, young children are uniquely at risk for development delays or impairments (12,13). Breastfeeding protects against childhood overweight and obesity, which are common causes of early onset of type 2 diabetes, but only 13% of babies are exclusively breastfed at the end of 6 months (14). The success rate among mothers who want to breastfeed can be improved through interpersonal, institutional, and policy support. Early child care providers also are in a unique position to support breastfeeding by ensuring that staff members at early child care centers are well-trained to meet national recommendations set by the American Academy of Pediatrics and outlined in *Caring for Our Children: National Health and Safety Performance Standards* (15) for supporting breastfeeding mothers. Support may include allowing mothers to breastfeed at the facility, feeding a mother’s pumped breast milk to her baby, thawing and preparing bottles of pumped milk as needed and keeping extra breast milk in a freezer. State and local jurisdictions can also set and enforce standards for early childhood care to ensure that standards are implemented (15). As of December 2011, only 6 states’ (Arizona, California, Delaware, Mississippi, North Carolina, and Vermont) licensing regulations contained language that met national recommendations for supporting breastfeeding (16).

Early child development and nutrition programs, such as the Special Supplemental Nutrition Program for Women, Infants, and Children (WIC) and the Child and Adult Care Food Program (CACFP), are federally funded food programs aimed at ensuring infants and children have access to nutritious food. WIC provides funds for buying healthful supplemental foods from WIC-authorized vendors, nutrition education, and help locating health care and other community services. CACFP provides meals and snacks to children and adults in day care facilities and in after-school programs. Although these federal programs help meet the daily nutritional needs of millions of young, low-income children during a critical period of growth and development, the dietary guidelines, structure, and reimbursements are outdated and complex, leaving many children without the benefits of these programs (17).

Evidence-based interventions that target young children are essential in ensuring healthy growth and development, including obesity prevention. *Healthy People 2020* (18) outlines several objectives and strategies to increase the proportion of persons aged 2 years or older whose diets are consistent with the US Department of Agriculture’s *Dietary Guidelines for Americans* (19). Many of these objectives could be achieved by enhancing the nutritional quality of food and beverages supported and supplied by federal nutrition assistance programs (20).

Several states, (eg, Delaware, North Carolina, Missouri, and Colorado) have implemented efforts to encourage improvement of meal standards associated with federal programs (21); however, effectiveness of these efforts are not well-defined and documentation of success from these programs is scant.

## Create Communities That Foster Health-Promoting Behaviors

Policy-level changes that influence the built environment can have a positive effect on the health of residents, particularly in low-resource communities. Environments with ample opportunities for residents to be physically active can enable adherence to physicians’ recommendations for exercise, and aspects of the built environment that affect physical activity and food behaviors are associated with obesity prevalence (22). According to the *Community Guide* (23), there is evidence to support the recommendation of community-scale and street-scale urban design and land-use policies to promote physical activity and overall health. These policies include community planning and development policies such as zoning codes that facilitate active transportation, connectivity of sidewalks and streets, and the provision for aesthetic and safety aspects of the physical environment (23). Improved access to public transportation is also a recommended strategy to increase physical activity within communities.

According to the Recommended Community Strategies and Measurements to Prevent Obesity in the United States from the Centers for Disease Control and Prevention (CDC) (24), promoting the availability of, and access to, affordable healthful food and beverages is recommended to improve community health. Of the 24 CDC recommendations, 7 are related to increasing the availability of healthful food in public venues and underserved areas (24). Food access policies such as those that provide incentives to food retailers to locate in underserved areas or to offer healthful food and beverage choices in those areas can reduce the barriers to improved nutrition as clinically recommended for health promotion and disease prevention. And although evidence of impact is emerging in adult populations (9), a systematic review reported that there is moderate evidence of the relationship between community and consumer nutrition environments and dietary intake of children and adolescents up to age 18 years (25). Other policies that can create sustainable nutrition improvements include provisions for farmers markets or farm-to-table initiatives and zoning laws that reduce the number of retail businesses or restaurants selling unhealthful foods within communities. Although there is a growing body of research on these policies, behavioral and disease prevention outcomes are often difficult to compare because of differences in assessment methods (9).

Both the built environment and food access depend on community and economic development policies within communities that determine zoning for residential, industrial, and commercial space, business activities, and resources such as schools and health centers. Considering health as a key component of policy decisions regarding community and economic development is consistent with the principles of “health in all policies” and the Health Impact Assessment (26). Informed by research on the social determinants of health, the Federal Reserve System and the Robert Wood Johnson Foundation have partnered to develop the Healthy Communities Initiative to highlight the need for closer coordination between the community development and health sectors, particularly in low-income communities (10).

## Broaden Health Care to Promote Health Outside the Medical System

Diabetes is a chronic disease that requires ongoing patient education, self-management, and clinical care to achieve desired outcomes. It is critical to understand how the patient’s ability to manage diabetes is affected by the Commission’s nonclinical vital signs such as employment, healthful food access, safe housing, and health literacy.

The PCMH is a mechanism for the redesign of health care delivery promoted through the Affordable Care Act. The PCMH model seeks to provide comprehensive, patient-centered, coordinated care that is accessible and has a consistent focus on quality improvement and patient safety.

The PCMH model incorporates some of the most successful strategies for improving glucose control, such as promotion of self-management, changes in the health care team, and case management, all documented in a meta-analysis to lower hemoglobin A1c (HbA1c) by approximately 0.5% to 0.6% (27). Numerous PCMH demonstration projects have been evaluated regarding cost, use, and quality metrics in diabetes care over the short term. Most have shown some reductions in cost, hospitalizations, and emergency department visits, although these may not be sustained and may not apply equally to those with type 1 diabetes and those with type 2 diabetes (28). PCMH demonstrations have also reported improvements in quality metrics such as HbA1c, low-density lipoprotein cholesterol, and blood pressure with improved patient and provider satisfaction; however, behavioral and psychosocial outcomes in these models are not well studied (29,30).

Although patients with low income and low education from racial/ethnic minority populations may benefit from the coordinated approach in a PCMH, many of these demonstrations have not targeted these groups. In a retrospective cohort study of 1,457 patients with diabetes receiving care in a PCMH academic practice, black patients were less likely to receive HbA1c testing, receive an influenza vaccination, or meet low-density lipoprotein cholesterol or blood pressure targets than non-Hispanic whites, after adjusting for other demographic, health, and socioeconomic factors (31). Black patients were also less likely to see their primary care provider during visits, less likely to see an endocrinologist, and more likely to be seen in the emergency department; however, there was no apparent difference in treatment intensity.

As part of the team-based approach that promotes patient self-management, community health workers (CHWs) are a key resource for connecting clinic to community, particularly in disadvantaged, underserved populations. CHWs are typically lay people from the community who are trained to serve as liaisons between patients and the health care community. They may work in teams with health care providers, provide group education in the community or clinical setting, or conduct home visits to follow up and address barriers to care. Regarding their role in diabetes care, interventions using nurse–CHW teams and CHWs trained as certified diabetes educators have been associated with mean HbA1c reductions of about 0.5% in numerous evaluations (32).

The Chronic Disease Self-Management Program (CDSMP) trains patients with chronic diseases as lay leaders in community settings to promote self-confidence in symptom control, decision-making, and patient–provider communication. A longitudinal study of the program among 1,170 participants demonstrated reductions in emergency department visits at 12 months and hospitalizations at 6 months as well as improved self-reported health, patient–physician communication, and medication compliance (33). Among patients with diabetes, results have been more variable. A trial with 196 participants recruited from 2009 through 2011 in a health care system in Texas found no significant differences between groups in HbA1c reduction over 12 months, although both groups saw HbA1c reductions from baseline of approximately 0.6% (34). This study also found no benefit to the CDSMP in diabetes self-care activities (34). However, in a separate analysis, patients in the CDSMP arm of this trial did have reduced odds of diabetes-related hospitalization or emergency department visits and longer times before hospitalization than the control arm (35). An uncontrolled longitudinal study of 114 patients found significant improvement in HbA1c at 6 months among patients with baseline HbA1c greater than 7% after participation in a CDSMP (36).

We highlight 2 successful interventions targeting underserved populations. These interventions seek to connect clinic to community.

### Project Sugar 2

Project Sugar 2, conducted in Baltimore, Maryland, randomized 542 African Americans in an urban managed-care organization during 2001 through 2003 to either biannual telephone counseling with a lay health educator and educational mailings or to an intensive intervention involving a nurse case manager and CHW visits. CHWs and case managers used clinical algorithms and intervention action plans, addressing topics ranging from nutrition and medication adherence to socioeconomic issues, to determine the frequency and intensity of follow-up and to maintain patient communication with health care providers. There was no significant difference in HbA1c between groups at 24 months after adjusting for age, baseline HbA1c level, and duration of follow-up. However, emergency department visits were significantly reduced in the intensive intervention arm by 23% at 24 months. At 24 and 36 months, those receiving the higher-intensity care (at least 2 visits from nurse case manager, or 4 visits from CHW, or both) saw the most benefit in reduction of emergency department visits (37).

### The South Side Diabetes Initiative

The South Side Diabetes Initiative in Chicago is an intervention, started in 2009, involving 6 health centers in a quality-improvement collaborative, patient activation, provider training, and community partnerships and outreach (38). The 6 health centers collectively serve just over 7,200 patients with diabetes annually. The quality-improvement collaborative shares best practices among health centers (eg, diabetes registries, case management, CHW interventions, and group medical visits). Patient activation tailors self-management education to literacy level and income restrictions. Providers are trained on cultural competency, behavioral counseling, and shared decision making. Finally, community outreach involves collaboration with local farmers markets, grocery stores, and food pantries to discount healthful food and provides education as well as medical home referrals. Although components have not been studied in aggregate, the quality-improvement collaborative enhanced perceived chronic care delivery, patient activation improved self-management behaviors and HbA1c levels, and provider training increased confidence in communication. Among 21 patients surveyed after receiving culturally tailored diabetes education and shared decision-making training, significant improvements were seen in self-reported dietary adherence, glucose monitoring, and foot care, and HbA1c declined from 8.24% at baseline to 7.33% at 3 months (*P* = .02) (39).

## Conclusion

Successful prevention and management of diabetes will require efforts that go beyond traditional clinical care, particularly in underserved and socially disadvantaged populations. There are evidence-based and promising strategies for intervening in early life, in the community, and at the nexus between the community and clinical settings. More research is needed to further establish the effectiveness of these approaches, particularly to determine the specific pathways through which clinical–community connections help to improve diabetes prevention and treatment outcomes. Identifying opportunities to intervene outside the examination room will be critical to effectively prevent and manage both obesity and diabetes. The Commission recommendations for health promotion offer a useful guide for areas to target. Policies are needed that support increased investments in 1) adequate nutrition through breastfeeding and other supports in early childhood, 2) community and economic development that includes health-promoting features of the physical, food, and social environments, and 3) evidence-based interventions that reach beyond the clinical setting to enlist community members in diabetes prevention and management.
